# Obesity and Eating Disturbance: the Role of TFEQ Restraint and Disinhibition

**DOI:** 10.1007/s13679-019-00365-x

**Published:** 2019-11-07

**Authors:** Eleanor J. Bryant, Javairia Rehman, Lisa B. Pepper, Elizabeth R. Walters

**Affiliations:** Division of Psychology, Faculty of Management, Law and Social Sciences, University of Bradford, Bradford, UK

**Keywords:** TFEQ, Restraint, Disinhibition, Obesity, Eating disturbance

## Abstract

**Purpose of Review:**

Literature from the past five years exploring roles of Three-Factor Eating Questionnaire (TFEQ) Restraint and Disinhibition in relation to adult obesity and eating disturbance (ED) was reviewed.

**Recent Findings:**

Restraint has a mixed impact on weight regulation, diet quality, and vulnerability to ED, where it is related detrimentally to weight regulation, diet, and psychopathology, yet can serve as a protective factor. The impact of Disinhibition is potently related to increased obesity, poorer diet, hedonically driven food choices, and a higher susceptibility to ED.

**Summary:**

Restraint and Disinhibition have distinct influences on obesity and ED and should be targeted differently in interventions. Further work is required to elucidate the mechanisms underlying TFEQ eating behavior traits.

## Introduction

It is clear that the susceptibility of some individuals to gain weight and be vulnerable to problematic eating behaviors in an obesogenic environment is an important issue. The prevalence of obesity has doubled since 1980, with worldwide estimates of 108 million children and 604 million adults considered obese in 2015 [1]. Moreover, globally, there were 4.0 million deaths related to obesity, reflecting the detrimental impact of obesity on health [1]. Such susceptibility within the obesogenic environment implies the presence of particular dispositions to either create a positive energy balance or to create behaviors which try to control energy balance in those affected. These dispositions can be measured psychometrically; over the past 30 years, the Three-Factor Eating Questionnaire (TFEQ) [2] has gained great popularity in measuring eating behavior dispositions or traits, which are related to weight regulation. Other eating behavior trait scales do exist, for example, the Restraint Scale [3] and the Dutch Eating Behavior Questionnaire [4]; however, the major strength of the TFEQ is the existence of vast evidence to suggest an important and robust role for the TFEQ traits in obesity, eating styles, eating disturbance, and associated factors, such as personality (e.g., Bryant et al. [5] for a review of Disinhibition and see Mills et al. [6] for a review of Restraint). The popularity of the TFEQ is due to the utility of the eating behavior traits that it measures, hence why these traits are the focus of this particular review.

The TFEQ assesses three factors; Restraint, Disinhibition, and Hunger. Restraint refers to an individual’s concern over weight control and strategies which are adopted to maintain body weight and restrict eating, for instance, using small portions, avoiding fattening foods, and stopping eating before reaching satiation, in order to limit food intake. Disinhibition reflects a tendency towards overeating and eating opportunistically in an obesogenic environment, for example, eating in response to negative affect, being unable to resist food cues, and overeating in response to the palatability of food. Hunger is concerned with the extent to which hunger feelings are perceived and the extent to which such feelings then evoke food intake. For example, intense feelings of hunger resulting in consumption in excess of three meals per day, feeling an absence of satiety, or creating unpleasant gastric sensations [2]

Further psychometric evaluation has revealed several sub-factors within the TFEQ (see Table 1). For example, Bond et al. [7] identified three Disinhibition sub-factors; Habitual, Situational, and Emotional susceptibility; three Restraint sub-factors of Strategic Dieting Behavior, Attitude to Self-Regulation, and Avoidance of Fattening Food; and two Hunger sub-factors of Internal and External Locus. Westenhoefer et al. [8] discovered Rigid and Flexible Restraint, and Niemeier et al. [9] suggested Internal and External Disinhibition. In addition, shorter revised versions of the TFEQ have also been created for adults [10, 11] and children [12, 13]. The revised versions of the TFEQ assess three factors: Cognitive Restraint (CR), as in the original TFEQ; Uncontrolled Eating (UE)—an amalgamation of the Disinhibition and Hunger scales of the original TFEQ which refers to responsivity to food palatability, social cues and hunger, resulting in eating episodes; and Emotional Eating (EE), referring to eating episodes elicited by negative affect.

**Table 1:** The Three-Factor Eating Questionnaire and psychometrically revised sub-factors

Author	Factors/Sub-factors	Explanation	Example
Stunkard and Messick (1985)	Restraint (21 items)	Restricting food intake to control body weight.	*I count calories as a conscious means of controlling my weight.*
	Disinhibition (16 items)	Opportunistic eating.	*I usually eat too much at social occasions, like parties and picnics.*
	Hunger (14 items)	Eating episodes elicited by feelings of hunger.	*I am always hungry enough to eat at any time.*
Westenhoefer et al. (1999)	Rigid Restraint (12 items)	An all or nothing approach to dieting.	*Do feelings of guilt about overeating help you to control your food intake?*
	Flexible Restraint (16 items)	A graded approach to dieting, whereby “forbidden” foods are allowed in moderation.	*While on a diet, if I eat food that is not allowed, I consciously eat less for a period of time to make up for it.*
Karlsson et al. (2000)—TFEQrR18	Cognitive Restraint (6 items)	Restricting food intake to control body weight.	*I deliberately take small helpings as a \means of controlling my weight.*
	Uncontrolled Eating (9 items)	Opportunistic and overeating eating elicited by palatability, hunger and social situations.	*Sometimes when I start eating, I can’t seem to stop.*
	Emotional Eating (3 items)	Eating episodes provoked by emotions.	*When I feel anxious, I find myself eating.*
Bond et al. (2001)	Restraint: Strategic Dieting Behavior (4 items)	Behaviors which are utilized to control body weight.	*I deliberately take small helpings as a way to control my weight.*
	Restraint: Attitude to Self-Regulation (5 items)	An individual’s perspective on eating and weight control	*I enjoy eating too much to spoil it by counting calories or watching my weight.*
	Restraint: Avoidance of Fattening Foods (4 items)	Behavioral techniques adopted to avoid high fat foods.	*I do not eat some foods because they make me fat.*
	Habitual Disinhibition (5 items)	Repeated episodes of eating / overeating.	*I start dieting in* *the morning, but because of any number of things that happen during the day, by evening I have given up and eat what I want, promising myself to start dieting again tomorrow.*
	Situational Disinhibition (5 items)	Eating episodes triggered by environmental cues.	*When I am with someone who is overeating, I usually overeat too.*
	Emotional Susceptibility (3 items)	Eating which is elicited by negative emotion.	*When I feel blue, I often overeat.*
	Internal Locus of Hunger (6 items)	Internal regulation and interpretation of hunger	*I get so hungry my stomach feels like a bottomless pit*
	External Locus of Hunger (6 items)	Hunger activated by external cues	*Being with someone who is eating often makes me hungry enough to eat also.*
Niemeier et al. (2007)	Internal Disinhibition (6 items)	Eating in response to internal thoughts and feelings.	*Sometimes things just taste so good that I keep on eating even when I am no longer hungry.*
	External Disinhibition (8 items)	Eating elicited by external, situational cues.	*Do you eat sensibly in front of others and splurge alone?*
Cappelleri et al. (2009)—TFEQR21	Cognitive Restraint (6 items)	Restricting food intake to control body weight.	*I consciously hold back on how much I eat at meals to keep from gaining weight.*
	Uncontrolled Eating (9 items)	Opportunistic and overeating eating elicited by palatability, hunger and social situations.	*I’m always so hungry that it’s hard for me to stop eating before finishing all of the food on my plate.*
	Emotional Eating (6 items)	Eating episodes provoked by emotions.	*When I feel tense or ‘wound up’, I often feel I need to eat.*

Previous work has demonstrated the important roles that these traits play in obesity and eating disturbance (e.g., see Bryant et al. [5] for a review); however, this evidential review is now dated. The aim of the current narrative review, therefore, is to explore the impact of the eating behavior traits, TFEQ Restraint and Disinhibition, upon obesity and eating disturbance, addressing literature from the last 5 years (2013–2018), in relation to adult body weight and obesity, disturbed and disordered eating, diet quality, cognitive profile, and weight loss.

## Method

In this review, we considered literature from the last 5 years which assessed TFEQ Restraint and Disinhibition, or revised versions of the TFEQ (CR, UE and EE), in adults (18 years or older) and their roles in relation to issues of obesity and body weight, eating behavior, cognitive profile, diet quality, disturbed and disordered eating, and associated issues.

A literature search was performed in PsycINFO, Science Direct, and PubMed; this was restricted to English language. Epidemiological, observational, and intervention studies (including randomized control trials) were included and only original articles were reviewed. Reference lists and forward citations of included studies were screened. Titles and abstracts were screened independently for eligibility (EB). Full-text articles were independently screened for inclusion by EB, JR, LBP, and ERW using the following inclusion criteria:

1. Use of an adult sample (18+ years).
2. Use of normal weight or overweight/obese samples
3. Studies which utilized the TFEQ to assess Restraint and Disinhibition.
4. Studies published within the last 5 years (2013–2018).

From the search, 76 papers were retained as eligible and reviewed. Papers were excluded if Restraint and/or Disinhibition were assessed using any other tool than the TFEQ, if adolescent or child samples were included, if the TFEQ was used in a study, but no results pertaining to Disinhibition or Restraint were reported, or if the paper was a review article. The papers were organized into sections depending upon the subject matter of the research, to look at the impact of eating behavior traits on body weight and obesity and on diet quality and cognitive profile, in relation to disturbed and disordered eating patterns and considering the impact of eating behavior traits on weight loss interventions.

## Eating Behavior Traits, Body Weight, and Obesity

Evidence suggests that as body mass index increases, Disinhibition and Hunger increase while Restraint decreases [14–17]. When considering Disinhibition (and UE and EE), there is a consistent representation that a higher level of Disinhibition is positively associated with BMI [18–21] as well as weight and fat mass [22, 23], while a higher UE [24], UE and EE [25, 26], and EE [26–29] are associated with an increased BMI. These data are supported by genetic studies which reveal that genes associated with increased BMI are also positively associated with UE and EE [27], suggesting eating behavior traits may be partially genetically determined [30–32]. In addition, parent-offspring eating behavior dyads indicate that parental Rigid Restraint and Disinhibition are related to a higher offspring BMI [33], suggesting that family environmental factors are also related to obesity transmission within families.

The evidence for Restraint is conflicting and proposes that Restraint is positively related to BMI [19, 27, 29, 34] where a higher level of Restraint increases the risk of obesity as much as fourfold [24] or where Restraint is lower in obese women [35], or not related to BMI at all [22, 25]. The mechanisms underlying why variations in the BMI and body composition of those with differing Disinhibition and Restraint scores exist are complex, and can at least be partially explained by diet quality and the eating patterns adopted by these individuals, for example, whether a flexible or rigid approach to Restraint is adopted, or depending upon the interaction between Disinhibition and Restraint and the subsequent impact of this on body weight.

## Eating Behavior Traits, Diet Quality, and Cognitive Profile

As expected, Disinhibition was found to be detrimentally associated with diet quality [14, 36] and self-determination in relation to eating behavior [37]. In addition, the amalgamated Disinhibition and Hunger factors of UE and EE are also positively associated with energy and fat intake [27], possessing a diagnosis of diabetes and responding to food insecurity in older adults [38]. Together, these suggest a behavioral profile which is detrimental to weight regulation and maintaining good health. Surprisingly, however, Disinhibition was not significantly related to eating in the absence of hunger in lean individuals [39], or was it related to snacking initiation [40]. The poorer dietary behavior of those with a higher Disinhibition (or UE) could be explained in part, by the higher impulsivity in relation to palatable foods possessed, regardless of their Restraint score [41], suggesting a more automatic response to food. Here, a cognitive profile emerges which supports a less healthful and more hedonically driven dietary response. For instance, those with a high Disinhibition show a higher liking [42], and wanting [18] of food, stronger food cravings [23, 43, 44], and a lower willingness to control eating behavior. In addition, they demonstrate a higher positive reinforcing value of food [45], show a vulnerability to accepting distressing thoughts (from negative reinforcement) [46], and have lower eating-related mindfulness (for those with a higher UE and EE) [47]. Indeed, collectively, this evidence supports the theoretical underpinning of Disinhibition, UE and EE, whereby those with higher scores are more motivated by the palatability of food, eat opportunistically, and are susceptible to eating in response to emotion as opposed to eating intuitively [2, 10, 11].

Responsiveness to emotion has also been found to impact diet quality and eating patterns in these individuals. Those with a higher UE and EE have a higher susceptibility to perceived stress [48] and a lower distress tolerance [49], and EE was associated with burnout in students [50]. Such responsiveness to negative emotions was also associated with lower eating competence, higher responsiveness to emotional and external cues to elicit eating episodes, and a poorer diet quality [48]. In addition to this, a higher level of Disinhibition was related to a negative self-evaluation and poorer eating regulation [51]. Unexpectedly, however, functional magnetic resonance imaging (fMRI) evidence suggests that Restraint, Disinhibition, and Hunger are not associated with brain regions connected with food cue reactivity [52]. These studies demonstrate how an eating behavior profile characterized by self-medicating negative affect can lead to poorer control over eating behavior and therefore a poorer weight regulation.

On the other hand, similar to the BMI evidence, the findings in relation to Restraint are mixed. It is possible that these mixed findings could reflect the type of Restraint exerting the most influence (e.g., Rigid or Flexible Restraint) on an individual; however, studies do not consistently report these Restraint sub-factors consistently to state this conclusively. Restraint was found to be related to a more healthful dietary profile [14, 36], a higher, but non-significant, satiety quotient [53], a lower energy intake [18], lower craving and liking of processed foods [54], a lower fat intake [27], and lower appetite ratings [55], all of which supports a behavioral profile conducive for weight regulation. This is in line with the behavioral tendencies measured by TFEQ Restraint. Restraint measures an individual’s cognitive effort to control their food intake to manage their body weight [2, 10]. Unlike other measures of Restraint, TFEQ Restraint only assesses efforts to regulate food intake and body weight, whereas other measures of Restraint, such as the Restraint Scale [3], also assess periodic disruption of this imposed restraint over food consumption, which leads to overeating episodes. Thus, it makes theoretical sense that those with a higher TFEQ Restraint engage in behaviors which favor a tighter regulation over energy intake and subsequently body weight, with a lower tendency to overeat. However, counter-intuitively, Restraint is also related to larger portion norms for men [56], predicts food intake from a test meal [57], and is not associated with compensatory behaviors to control for energy intake [58]. Furthermore, no effect of Restraint on consumption was found when portion size was altered [59] or in adherence to a dietary intervention [60].

This presents a complex picture of the impact of Restraint on dietary composition. A possible explanation for these differences could be derived from the goal conflict theory [61] which suggests weight regulation issues result from the conflict between the goal of weight control and the goal of eating enjoyment; the goal of weight control is often subjugated by the hedonic expectation of food [62]. The current obesogenic environment presents a plethora of palatable foods and so the goal of eating enjoyment is more often primed, meaning that a higher cognitive effort is required to maintain the goal of weight control [61]. This cognitive effort is likely to be particularly challenging when other external factors are present (for example, relationships, work-life balance, emotional fatigue) which may reduce the individual’s cognitive capacity to review and control their food intake, potentially leading to the goal of eating enjoyment being more easily prioritized [61]. Consequently, this then results in unhealthy eating patterns and weight gain [63]. Indeed, supporting evidence suggests that attempts at Restraint can create food cravings, which increases the risk of further weight gain and obesity [64]. Here, higher Restraint led to a higher food craving, which was subsequently related to a higher UE and EE [64].

## Eating Behavior Traits and Disordered and Disturbed Eating

Both Disinhibition and Restraint are found to be related to the psychopathology of disturbed eating behavior and eating disorders. Disinhibition appears to be a behavioral indicator of a loss of control over eating, where an individual consumes greater quantities of food, independently of their level of Restraint [65]. This is reflected in those who have a binge eating disorder (BED) diagnosis possessing a higher Disinhibition and Hunger [66]. Increases in objective and subjective binge eating and objective overeating are also observed with higher levels of Disinhibition [67], whereas Restraint has been associated with objective binge eating but not overeating [67].

Furthermore, Disinhibition can predict excessive consumption and objective binge eating episodes before and after fasting periods, where eating disorder risk has been associated with fasting behaviors (complete fasting for 24 hours) [68]. Disinhibition and self-reported, voluntary fasting frequency predicted positive shifts in post-fast body image [69] suggesting that self-imposed food restriction can have a positive reinforcement impact on non-clinical samples. Overall, however, these studies suggest that Disinhibition is related not only to eating disorder susceptibility but also to more pathological symptomatology within disordered eating.

This is corroborated with evidence from those who score highly on the Yale Food Addiction Scale, reporting higher levels of Disinhibition, a high tendency to overeat [70, 71], and a lower distress tolerance [49], which exacerbates their overeating response. In concordance, evidence also suggests that disordered and disturbed eating behaviors are similarly related to higher depression and Disinhibition levels [23]. Indeed, CR, UE, and EE have been related to higher neuroticism, anxiety, and depression scores, which were also associated with dysfunctional eating patterns [72], weight stigma, and weight bias internalization [73]. Significant feelings of guilt and food craving have also been reported by those with a high Disinhibition [44] and high EE [35], where the craving was subsequently related to binge eating and obesity. Furthermore, Disinhibition has been found to be negatively associated with intuitive eating and body appreciation [74]. Thus, a pattern of behaviors associated with disturbed eating patterns and poorer mental health for those with a high Disinhibition emerges, which is compounded by a lower tolerance to adverse emotional states.

On the other hand, Restraint is used as a means to control leanness to strive towards body image ideals [75]; here, neuroticism partially mediates the associations between Restraint, body dissatisfaction, and binge eating [76]. The association of Restraint with higher eating disordered attitudes is not always expressed in energy restriction; evidence suggests that in female students, Restraint resulted in sub-optimal energy consumption relative to physiological need as a result of higher physical activity rather than energy restriction [28]. In support of this, Linardon and Mitchel [74] found that both Flexible and Rigid Restraint predicted exercising for weight loss and body checking, while Rigid Restraint predicted over-evaluation of weight and shape. In this study, Restraint was found to be a strong predictor of both disordered eating and negative body image [74]. Surprisingly, no relationship between mindful eating and Restraint has been found [34]. However, a negative relationship between Restraint and intuitive eating existed, which predicted higher disordered eating levels [34] Restraint as negatively associated with intuitive eating is not unexpected, since Restraint relies on cognitive effort to restrict food intake, rather than relying on internal satiety signals to control energy intake. This is in line with evidence which suggest a reliance on increased physical activity to control body weight in Restrained individuals, supporting more external, than internal, control imposed on energy restriction [28].

## Eating Behavior Traits and Weight Loss Interventions

The eating behavior trait profiles of individuals undergoing weight loss intervention are clearly of high importance, as the eating behavior response of an individual can determine their weight loss success. There are robust findings that in following weight loss interventions, a change in eating behavior traits are seen, whereby there is an increase in Restraint [77, 78] [52, 79, 80] and a decrease in Disinhibition (or UE) [81, 82] [52, 79, 80] and Hunger [83–87]. However, one study found no change in TFEQ factors despite significant weight loss [60]. This could be a result of the dietary intervention imposed which required participants to adhere to various vegetarian and vegan diets. In comparison, those studies which utilized, for example, the Mediterranean diet [83] and high-protein and high0carbohydrate diets [79], calorie restricting diets [88], and lifestyle intervention including dietary advice [80] saw increases in TFEQ Restraint and decreases in Disinhibition. Weight loss with these diets was associated with increases in Restraint [80, 88], decreases in Disinhibition [79], or both [83]. It is plausible that changes in eating behavior traits are associated with changes in appetite peptides, where TFEQ Hunger positively predicted ghrelin levels during weight loss [85]. Even though Disinhibition and Restraint did not reach significance in this study, further work needs to be done to explore the relationship between TFEQ, weight loss, and weight loss maintenance. Whether the weight loss itself leads to changes in eating behavior traits, or whether changes in eating behavior traits cause weight loss, or an interaction between the two requires further elucidation.

However, evidence does suggest that weight loss success is related to changes in eating behavior traits, whereby an increase in Flexible Restraint and decrease in Rigid Restraint is related to greater weight loss [89], and where a reduction in Disinhibition predicts weight loss over a 12-month period [79]. In addition, decreases in Restraint and increases in Disinhibition are found to be associated with weight regain over 10 years [90]. The TFEQ eating behavior traits do not act in isolation, for example, even though Restraint was the best predictor of weight reduction, the effect of Restraint on changes in weight and anthropometry was found to be moderated by increases in Disinhibition [91]. Further work examining the interaction between the eating behavior traits on weight loss success is warranted.

Bariatric surgery candidates are found to have higher Disinhibition and Hunger scores [92], although this is not always the case when comparing surgical with non-surgical candidates [93]. Following surgery, a robust finding is that Disinhibition (or UE and EE) decreases, alongside either an increase in Restraint [84, 94–96] or no change in Restraint [97–100], thus indicating a change in eating behavior traits which favors weight regulation. Baseline scores usually show limited ability in predicting post-surgery weight loss success. However, evidence suggests that baseline Restraint negatively impacts post-surgical weight loss, while baseline Disinhibition is positively correlated with weight reduction [93, 101]. This suggests that bariatric surgery is effective in improving the problematic eating patterns displayed by those with a high Disinhibition, which results in a greater weight loss. There is evidence that changes in eating behaviors post-surgery are more strongly associated with weight loss success. Those who lose more weight notice a greater reduction in Disinhibition and Hunger between 1 and 10 years post-surgery [94] or see an increase in Restraint and a decrease in UE and EE [102]. Weight loss success is therefore related to those who show an eating behavior profile which favors body weight regulation [95]. Therefore, eating behavior traits make a potent contribution to weight loss success following bariatric surgery. Despite this, a study by Parker et al. [103] evaluated various measures of disordered eating (including the TFEQ) in bariatric surgery patients; they concluded that none of the original measures evaluated were suitable for this cohort in their original forms. The authors concluded that the Eating Disorder Examination Questionnaire was the most suitable measure for use in pre- and post-operative scenarios.

## Conclusion

**Fig. 1:**
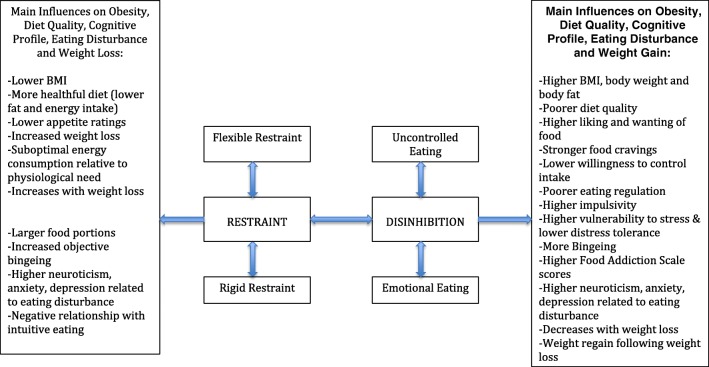
Summary of the influence Restraint and Disinhibition have upon obesity, diet quality, cognitive profile, eating disturbance, and weight loss

This narrative review has demonstrated that both Disinhibition and Restraint play an important role in obesity status, diet quality, and on the psychopathology of disturbed and disordered eating behaviors (Fig. 1). Due to the observational nature of many of the studies included here, a causal relationship between these eating behavior traits, obesity, and eating disturbance cannot be inferred. However, understanding what characterizes individuals with high scores on these eating behavior traits is important, as such insight could serve to improve interventions for weight loss and for improving eating disorder symptomatology. For instance, those with a high Disinhibition are more vulnerable to having an increased BMI and fat mass, poor diet quality, poorer health, more problematic eating behaviors, weight regain following weight loss, and more binge eating, food craving, and food addiction. Conversely, the impact of Restraint is mixed. On one hand, Restraint is related to a lower body weight, better weight regulation, and a better diet quality. On the other hand, Restraint is additionally related to a susceptibility to obesity, a poorer diet, and overeating. Where the impact of Disinhibition on obesity is potent in increasing energy intake and susceptibility to disturbed eating, the action of cognitive Restriction produces differential results, perhaps due to difficulty in maintaining cognitive control in an obesogenic environment replete with palatable foods. This complexity suggests that intervention should target reducing Disinhibition, rather than increasing Restraint.

Furthermore, the association of TFEQ Restraint and Disinhibition with the psychopathology of disturbed and disordered eating is also of great importance. High levels of Disinhibition are robustly related to a higher susceptibility to disordered eating and more pathological symptomatology of eating disorders. High Disinhibition individuals also have higher levels of adverse behaviors and thought patterns associated with disturbed eating, such as internalization of weight bias and stigma, lower body appreciation and guilt, and further increasing susceptibility to eating disorders, particularly when coupled with a lower tolerance of distress. TFEQ Restraint is also related to increased levels of disturbed and disordered eating behaviors and associated negative behaviors. Studies, which assess eating disorders, more often use the Eating Disorder Inventory measure of Restraint, rather than the TFEQ Restraint, explaining the relatively few studies included here which examine disordered eating. However, the evidence presented suggests that the TFEQ Restraint is effective in identifying problematic and disturbed eating behavior in clinical samples. Specific targeting of the reduction of Disinhibition and regulation of Restraint (improving Flexible Restraint and decreasing Rigid Restraint) within interventions to counteract disturbed and disordered eating is warranted[104].

In addition, it is important to note that scores on the TFEQ factors tend to be higher in females than males [15, 17, 19, 64, 105] suggesting differences in eating behavior traits between men and women. However, Leblanc et al. [37] found few differences between the sexes on the TFEQ, with women only scoring significantly higher in Emotional Susceptibility to Disinhibition. Furthermore, older individuals are found to have higher Restraint [59, 105] and lower Disinhibition [17, 27, 105]. It is unclear why these differences exist between men and women, and over age categories, and further exploration is necessary. However, these differences are of consequence, particularly when designing interventions to combat obesity or disturbed and disordered eating behaviors, as these approaches may need to be more specifically tailored. In addition, much of the research uses samples comprised mainly of women; leaving paucity in TFEQ research regarding men, and perhaps providing a skewed picture of the impact of TFEQ eating behavior traits on obesity and disturbed and disordered eating.
